# Development and validation of an in-hospital mortality risk prediction model for patients with severe community-acquired pneumonia in the intensive care unit

**DOI:** 10.1186/s12890-023-02567-5

**Published:** 2023-08-17

**Authors:** Jingjing Pan, Wei Bu, Tao Guo, Zhi Geng, Min Shao

**Affiliations:** 1 Department of Critical Care Medicine, The First Affiliated Hospital of Anhui Medical University, Hefei, China; 2 Department of Respiratory Intensive Care Unit, Anhui Chest Hospital, Hefei, China; 3 Center for Biomedical Imaging, University of Science and Technology of China, Hefei, China; 4 Department of Neurology, The First Affiliated Hospital of Anhui Medical University, Hefei, China; 5 Anhui Province Key Laboratory of Cognition and Neuropsychiatric Disorders, Hefei, China; 6 Collaborative Innovation Center of Neuropsychiatric Disorders and Mental Health, Hefei, China

**Keywords:** Severe community-acquired pneumonia, Intensive care unit, Mortality risk prediction, Nomogram

## Abstract

**Background:**

A high mortality rate has always been observed in patients with severe community-acquired pneumonia (SCAP) admitted to the intensive care unit (ICU); however, there are few reported predictive models regarding the prognosis of this group of patients. This study aimed to screen for risk factors and assign a useful nomogram to predict mortality in these patients.

**Methods:**

As a developmental cohort, we used 455 patients with SCAP admitted to ICU. Logistic regression analyses were used to identify independent risk factors for death. A mortality prediction model was built based on statistically significant risk factors. Furthermore, the model was visualized using a nomogram. As a validation cohort, we used 88 patients with SCAP admitted to ICU of another hospital. The performance of the nomogram was evaluated by analysis of the area under the receiver operating characteristic (ROC) curve (AUC), calibration curve analysis, and decision curve analysis (DCA).

**Results:**

Lymphocytes, PaO2/FiO2, shock, and APACHE II score were independent risk factors for in-hospital mortality in the development cohort. External validation results showed a C-index of 0.903 (95% CI 0.838–0.968). The AUC of model for the development cohort was 0.85, which was better than APACHE II score 0.795 and SOFA score 0.69. The AUC for the validation cohort was 0.893, which was better than APACHE II score 0.746 and SOFA score 0.742. Calibration curves for both cohorts showed agreement between predicted and actual probabilities. The results of the DCA curves for both cohorts indicated that the model had a high clinical application in comparison to APACHE II and SOFA scoring systems.

**Conclusions:**

We developed a predictive model based on lymphocytes, PaO2/FiO2, shock, and APACHE II scores to predict in-hospital mortality in patients with SCAP admitted to the ICU. The model has the potential to help physicians assess the prognosis of this group of patients.

## Introduction

Pneumonia is an acute respiratory infection classified into community-acquired pneumonia (CAP) and hospital-acquired pneumonia (HAP) [1]. CAP is an acute disease caused by a pulmonary parenchymal infection acquired outside the hospital. More than 1.5 million adults in the United States are hospitalized yearly for CAP, with high hospitalization costs [2]. Patients with mild to moderate CAP are admitted to the outpatient clinic, ward, or intensive care unit (ICU) for treatment, depending on severity. Severe CAP (SCAP) or CAP requiring ICU hospitalization has a significantly worse prognosis [3–7]. A secondary analysis of a population-based cohort study of hospitalized SCAP patients in the United States by Cavallazzi et al. found that 23% of patients required ICU admission and that the risk for SCAP patients admitted to the ICU was 17% in-hospital mortality and nearly 50% 1-year mortality [2].

In previous studies on the prognosis of SCAP, either the study population was outside the ICU [8–10], the risk factors for mortality were not readily available clinically [9, 10], no relevant predictive model was developed [11], or the model developed was not externally validated [10, 12]. Generally, only a few comprehensive and accurate evaluation systems can adequately assess the prognosis of patients with SCAP admitted to the ICU at this stage. Therefore, developing a mortality prediction model for such patients is necessary to identify the risk factors for death at an early stage. In addition, ICU physicians can improve patient management and make better decisions with the help of a reasonable model.

In our study, we analyzed clinical data of patients with SCAP admitted to the ICU retrospectively, developed a mortality risk prediction model using easily accessible variables, and performed external validation to identify patients with high mortality risk as early as possible. We anticipate that the results of this study will assist ICU physicians in their efforts to improve the prognosis of patients with SCAPs.

## Methods

### Study design

We conducted a two-center retrospective observational cohort study. The development cohort included 455 patients with SCAP from the Department of Critical Care Medicine of The First Affiliated Hospital of Anhui Medical University of China between June 2019 and October 2022. The validation cohort included 88 patients with SCAP admitted to the Department of Respiratory ICU at Anhui Chest Hospital, China, between July 2021 and November 2022. We retrospectively analyzed the clinical data of patients from these two cohorts. We established a mortality prediction model based on the development cohort. Model visualization was done using a nomogram. The validation cohort was substituted in the model for external verification.

#### Informed consent

of patients was waived by the First Affiliated Hospital Ethics Committee of Anhui Medical University because this study was retrospective. This study was approved by the First Affiliated Hospital Ethics Committee of Anhui Medical University. We conducted the study in strict compliance with the Declaration of Helsinki.

### Patients

The inclusion criterion for this study was ICU inpatients who met the diagnostic criteria for SCAP. Following the guidelines of the Infectious Diseases Society of America/American Thoracic Society, SCAP was defined as the fulfillment of 1 main diagnostic criterion or ≥ 3 minor diagnostic criteria [13, 14]. The main diagnostic criteria included the following: (1) respiratory failure requiring invasive mechanical ventilation and (2) septic shock requiring vasopressors even after fluid resuscitation. The minor diagnostic criteria included the following: (1) Respiratory rate > 30 breaths/min, (2) PaO2/FiO2 < 250 mmHg, (3) multilobar infiltration, (4) consciousness impairment and/or orientation disorders, (5) blood urea nitrogen > 20 mg/dL, (6) count of white blood cell < 4 × 10^9^ cells/L, (7) count of platelet < 100 × 10^9^ platelets/L, (8) central body temperature < 36 °C, (9) hypotension (Systolic blood pressure < 90 mmHg, requiring active fluid resuscitation). The exclusion criteria were as follows: (1) patients with considerable missing clinical information, (2) diagnosis of hospital-acquired pneumonia, and (3) uncertain outcomes.

### Data collection

The following clinical data were collected from the critical care electronic data mart within 48 h of admission to ICU: (1) demographic characteristics: sex, age; (2) chronic underlying disease comorbidities: hypertension, diabetes, tumors; (3) laboratory examinations: C-reactive protein (CRP), procalcitonin (PCT), neutrophils, lymphocytes, fibrinogen, D-dimer, blood urea nitrogen, total bilirubin, albumin, blood glucose, blood lactate, PaCO2, PaO2/FiO2; (4) severity of disease: whether combined with shock, APACHE II score, SOFA score; (5) clinical interventions: days of invasive mechanical ventilation, use of glucocorticoids. Patients admitted to the ICU with SCAP were divided into survival and death groups according to the in-hospital outcomes.

### Statistical analysis

We excluded the variables with missing values greater than 20%, and the remaining variables were supplemented based on the multiple imputation method. Thereafter, we performed descriptive statistics for both cohorts and compared the differences between them. The mean ± standard deviation or median (interquartile range) was used for continuous variables, while frequencies and proportions were used as the descriptive statistics for categorical variables. The normal distribution of each variable was examined using the Kolmogorov–Smirnov test. Continuous variables were compared using the Student’s t-test or Mann–Whitney U-test. Categorical variables were analyzed using either Fisher’s exact test or Pearson’s χ^2^ test.

The developmental cohort was divided into survival and death groups based on the occurrence of death during hospitalization as the outcome variable. Potential variables with P < 0.05 in the univariate logistic regression analysis were added to the multivariate analysis to identify the independent risk factors for hospital morbidity. Odds ratios (ORs) and 95% confidence intervals (CIs) were used to present the results.

A nomogram was developed based on these independent risk factors in the development cohort to predict the probability of death. The model of the nomogram was evaluated using the consistency index (C-index) for the validation cohort. In the development and validation cohorts, predictive models were assessed using the area under the receiver operating characteristic (ROC) curve (AUC), calibration curve analysis, and decision curve analysis (DCA).

Statistical analysis was performed using SPSS version 26.0 and R version 4.12. P < 0.05 for both bilaterals was considered statistically significant.

## Results

### Basic demographic characteristics

Descriptions of the clinical data of the development and validation cohorts are provided in Table 1. The development cohort was composed of 455 patients with SCAP, of which 295 (64.84%) were male, and 160 (35.16%) were female, with an average age of (61.93 ± 17.77) years. They were divided into 358 patients in the survival group and 97 patients in the death group according to their prognosis, with a mortality rate of 21.32%. The validation cohort included 88 patients with SCAP, there were 70 males (79.55%) and 18 females (20.45%) among them, and the average age was (67.49 ± 14.02) years. According to their prognosis, the validation cohort was divided into 68 patients in the survival group and 20 cases in the death group, with a mortality rate of 22.73%. There were no significant differences between the development and validation cohorts with respect to the mortality rate (P > 0.05).

**Table 1:** Comparison of clinical statistics between the development cohort and the validation cohort

Clinic data	Development cohort (n = 455)	Validation cohort (n = 88)	P-value
Age(year)	61.93 ± 17.77	67.49 ± 14.02	0.006
Sex			0.007
male	295 (64.84%)	70 (79.55%)	
female	160 (35.16%)	18 (20.45%)	
Hypertension			0.005
No	291 (63.96%)	70 (79.55%)	
Yes	164 (36.04%)	18 (20.45%)	
Diabetes			0.326
No	372 (81.76%)	68 (77.27%)	
Yes	83 (18.24%)	20 (22.73%)	
Tumors			< 0.001
No	439 (96.48%)	54 (61.36%)	
Yes	16 (3.52%)	34 (38.64%)	
CRP(mg/L)	104.84 ± 70.29	134.21 ± 92.80	< 0.001
PCT(ng/ml)	10.41 ± 24.61	7.22 ± 21.67	0.001
Neutrophils(10^9/L)	12.26 ± 7.12	11.18 ± 5.58	0.181
Lymphocytes(10^9/L)	1.07 ± 0.92	0.72 ± 1.00	0.002
Fibrinogen(g/L)	4.68 ± 1.97	4.63 ± 1.53	0.818
D-dimer(mg/L)	8.07 ± 9.56	1.78 ± 3.45	< 0.001
Urea nitrogen(mmol/L)	15.77 ± 11.37	309.05 ± 176.72	< 0.001
Total bilirubin(umol/L)	29.30 ± 46.29	16.39 ± 10.20	0.010
Albumin(g/L)	33.56 ± 6.18	28.93 ± 5.10	< 0.001
Blood glucose(mmol/L)	11.63 ± 5.24	9.89 ± 4.39	0.004
Blood lactate(mmol/L)	4.85 ± 12.33	3.59 ± 6.75	0.354
PaCO2(mmHg)	45.43 ± 15.63	48.78 ± 24.64	0.099
PaO2/FiO2	247.17 ± 96.46	144.99 ± 53.66	< 0.001
Shock			< 0.001
No	178 (39.12%)	57 (64.77%)	
Yes	277 (60.88%)	31 (35.23%)	
APACHE II score	21.83 ± 8.36	21.77 ± 7.30	0.950
SOFA score	8.37 ± 3.99	6.60 ± 2.73	< 0.001
ICU MV time(day)	7.49 ± 10.40	5.83 ± 5.10	0.145
Glucocorticoids			0.016
No	197 (43.30%)	26 (29.55%)	
Yes	258 (56.70%)	62 (70.45%)	
Death			0.769
No	358 (78.68%)	68 (77.27%)	
Yes	97 (21.32%)	20 (22.73%)	

Abbreviations: CRP, C-reactive protein; PCT, procalcitonin; PaCO2, arterial carbon dioxide partial pressure; PaO2, arterial oxygen partial pressure; FiO2, inhaled oxygen concentration; APACHE, Acute Physiology And Chronic Health Evaluation; SOFA, Sequential Organ Failure Assessment; ICU, Intensive Care Unit; MV, mechanical ventilation

### Construction of the predictive model

The results of the univariate regression analysis and the multivariate regression analyses for the development cohort are summarized in Table 2, which provides a detailed summary of the ORs and 95% CIs. It was observed that 23 variables were filtered using univariate logistic regression analysis to identify 12 statistically significant variables. These included CRP, PCT, lymphocyte, D-dimer, urea nitrogen, total bilirubin, blood glucose, PaCO2, PaO2/FiO2, Shock, APACHE II score, and SOFA score. Further multivariate regression analysis of these 12 variables showed that lymphocyte count, PaO2/FiO2, shock, and APACHE II score were independent risk factors for death in patients with SCAP admitted to the ICU in the development cohort. A one-unit lymphocyte increase within 48 h of admission was associated with approximately two-fold in-hospital mortality (OR: 1.996). A high APACHE II score was also a risk factor for in-hospital mortality (OR: 1.135). Shock can increase the risk of in-hospital death by approximately four-fold with a one-unit increase (OR: 4.093). PO2/FiO2 was a significant protective factor against in-hospital mortality because the OR was < 1 (OR : 0.997).

**Table 2:** Univariable and multivariable logistic regression between survivors and nonsurvivors in the development cohort

Variables	Univariable logistic regression			Multivariable logistic regression	
	OR (95% CI)	P value		OR (95% CI)	P value
Age	1.00 (0.99, 1.01)	0.9056			
Gender	0.99 (0.62, 1.59)	0.9790			
Hypertension	0.95 (0.59, 1.51)	0.8185			
Diabetes	0.78 (0.42, 1.44)	0.4254			
Tumors	0.85 (0.24, 3.03)	0.7986			
CRP(mg/L)	1.00 (1.00, 1.01)	0.0267		1.004 (1.000, 1.008)	0.060
PCT(ng/ml)	1.01 (1.00, 1.02)	0.0042		0.998 (0.987, 1.008)	0.691
Neutrophils(10^9/L)	1.03 (1.00, 1.06)	0.0517			
Lymphocytes(10^9/L)	1.93 (1.50, 2.48)	< 0.0001		1.996 (1.452, 2.744)	< 0.001
Fibrinogen(g/L)	0.99 (0.88, 1.11)	0.8328			
D-dimer(mg/L)	1.05 (1.02, 1.08)	0.0003		1.017 (0.983, 1.053)	0.321
Urea nitrogen(mmol/L)	1.02 (1.00, 1.04)	0.0335		0.992 (0.964, 1.021)	0.599
Total bilirubin(umol/L)	1.00 (1.00, 1.01)	0.0247		1.00 (0.994, 1.006)	0.947
Albuming(g/L)	0.99 (0.95, 1.02)	0.4283			
Blood glucose(mmol/L)	1.10 (1.05, 1.15)	< 0.0001		1.057 (0.999, 1.119)	0.054
Blood lactate(mmol/L)	1.01 (1.00, 1.03)	0.0983			
PaCO2(mmHg)	1.03 (1.01, 1.04)	< 0.0001		1.013 (0.994, 1.031)	0.180
PaO2/FiO2	1.00 (0.99, 1.00)	0.0011		0.997 (0.994, 1.000)	0.041
Shock	8.74 (4.27, 17.90)	< 0.0001		4.093 (1.807, 9.269)	0.001
APACHE II score	1.17 (1.13, 1.22)	< 0.0001		1.135 (1.083, 1.189)	< 0.001
SOFA score	1.20 (1.13, 1.27)	< 0.0001		0.999 (0.910, 1.096)	0.982
ICU MV time(day)	1.01 (0.99, 1.03)	0.3713			
Glucocorticoids	1.00 (0.64, 1.57)	0.9996			

Abbreviations: CRP, C-reactive protein; PCT, procalcitonin; PaCO2, arterial carbon dioxide partial pressure; PaO2, arterial oxygen partial pressure; FiO2, inhaled oxygen concentration; APACHE, Acute Physiology And Chronic Health Evaluation; SOFA, Sequential Organ Failure Assessment; ICU, Intensive Care Unit; MV, mechanical ventilation

**Fig. 1:**
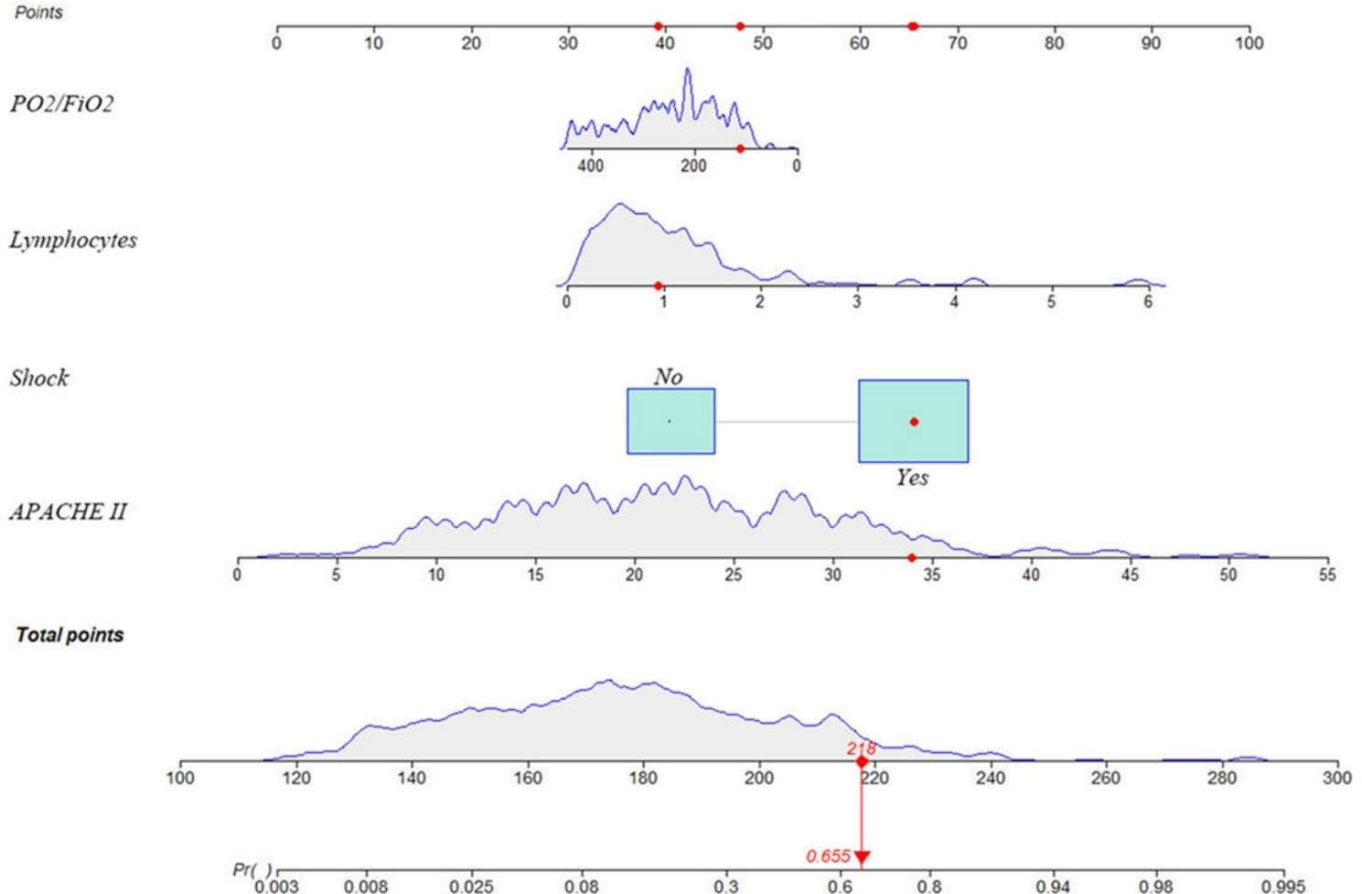
The nomogram for predicting the risk of mortality in SCAP patients admitted to the ICU. PaO2, arterial oxygen partial pressure; FiO2, inhaled oxygen concentration; APACHE, Acute Physiology And Chronic Health Evaluation

These variables were used to build a predictive model. A nomogram (Fig. 1) was constructed to predict the in-hospital mortality of patients admitted to the ICU with SCAP. The points corresponding to each variable were summed to obtain the total score and generate predicted mortality probability.

### Evaluation of the predictive model

**Figs. 2:**
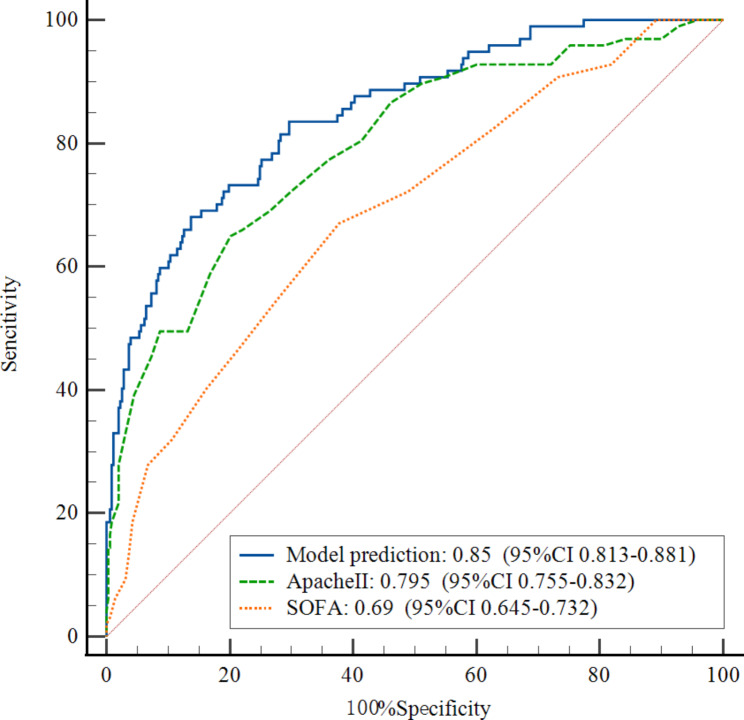
The receiver operating characteristic(ROC) curve of the predictive model, APACHE II and SOFA scoring systems for the development cohort. Model AUC was 0.850 (95% CI 0.813–0.881), APACHE II score 0.795 (95% CI 0.755–0.832), and SOFA score 0.69 (95% CI 0.645–0.732)

**Fig. 3:**
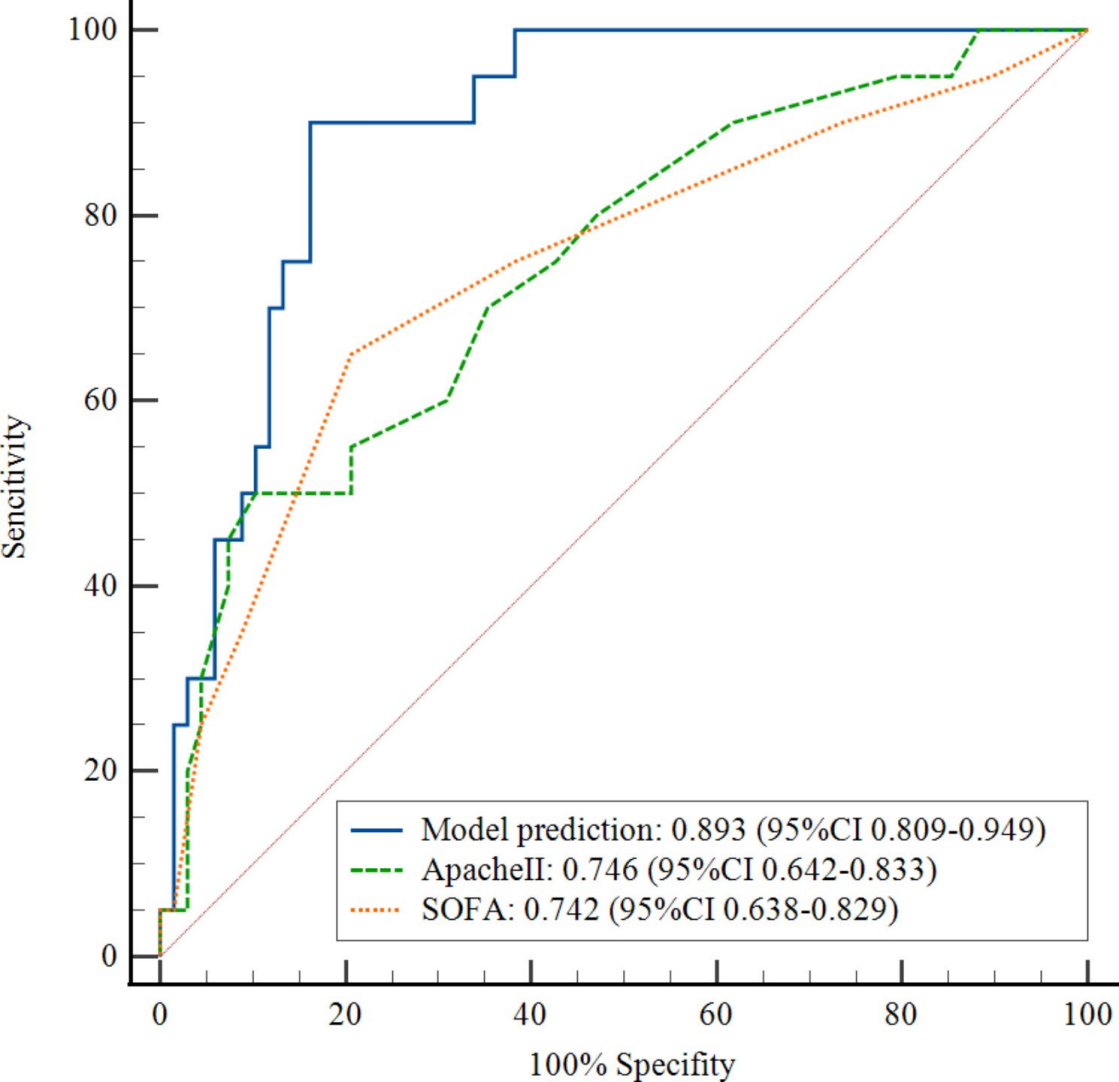
The receiver operating characteristic(ROC) curve of the predictive model, APACHE II and SOFA scoring systems for the validation cohort. Model AUC was 0.893 (95% CI 0.809–0.949), APACHE II score 0.746 (95% CI 0.642–0.833), and SOFA score 0.742 (95% CI 0.638–0.829)

The clinical data of the validation cohort, which included 88 patients with SCAP from another hospital, were incorporated into the prediction model for external validation. The external validation results showed that the C index of the validation cohort was 0.903 (95% CI 0.838–0.968). We used ROC curves to compare the model with two classical scoring systems (APACHE II and SOFA). Figure 2 showed the ROC curve for the development cohort, the AUC of our predictive model was 0.850 (95% CI 0.813–0.881), APACHE II score 0.795 (95% CI 0.755–0.832), and SOFA score 0.69 (95% CI 0.645–0.732). The ROC curve for the validation cohort was shown in Fig. 3, the AUC of the predictive model was 0.893 (95% CI 0.809–0.949), APACHE II score 0.746 (95% CI 0.642–0.833), and SOFA score 0.742 (95% CI 0.638–0.829). The results indicated that the model had well-predicted power and accuracy for SCAP mortality. Meanwhile, it outperformed the traditional APACHE II and SOFA scoring systems.

**Figs. 4:**
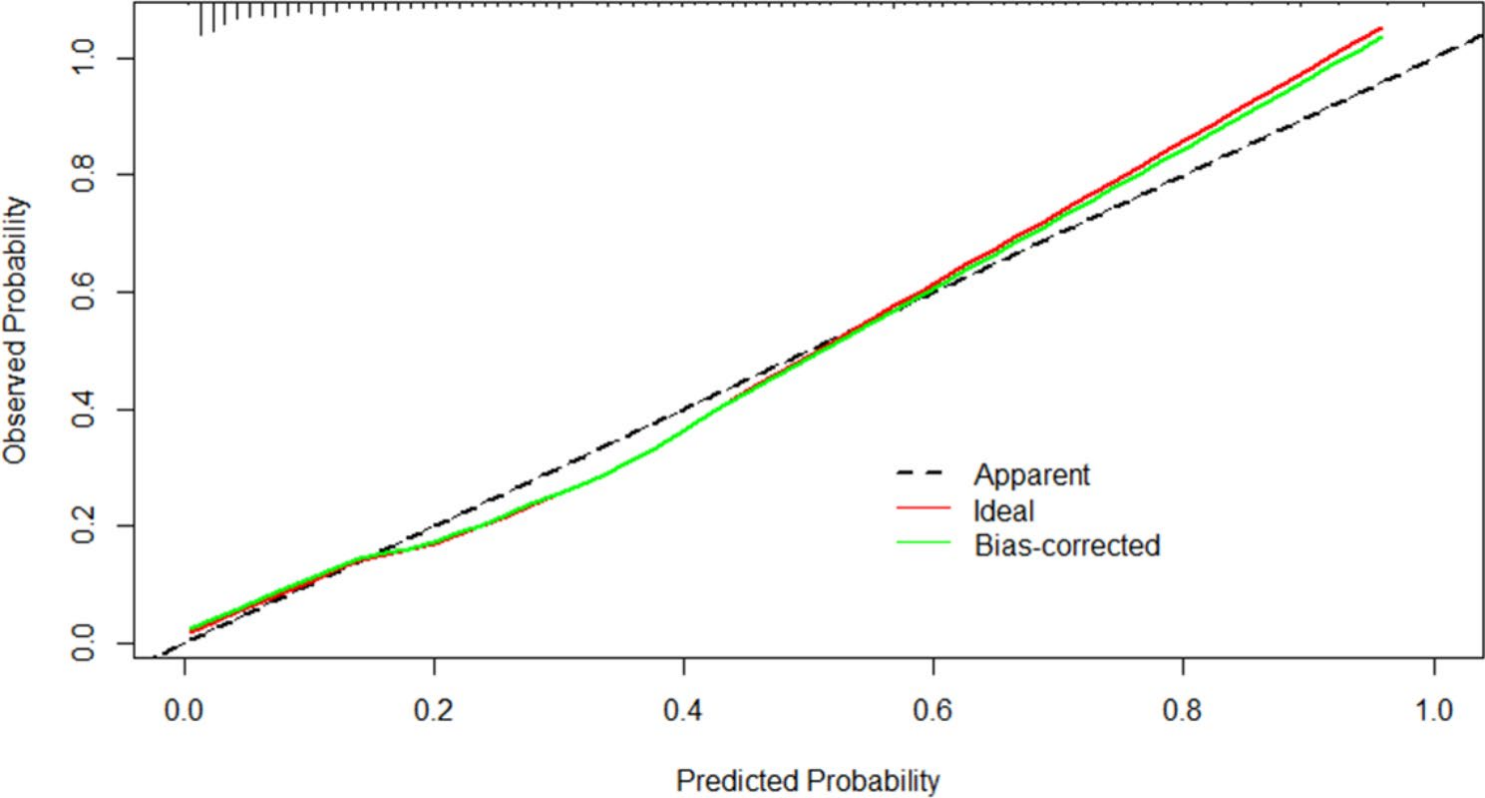
The calibration curve of nomogram in the development cohort

**Figs. 5:**
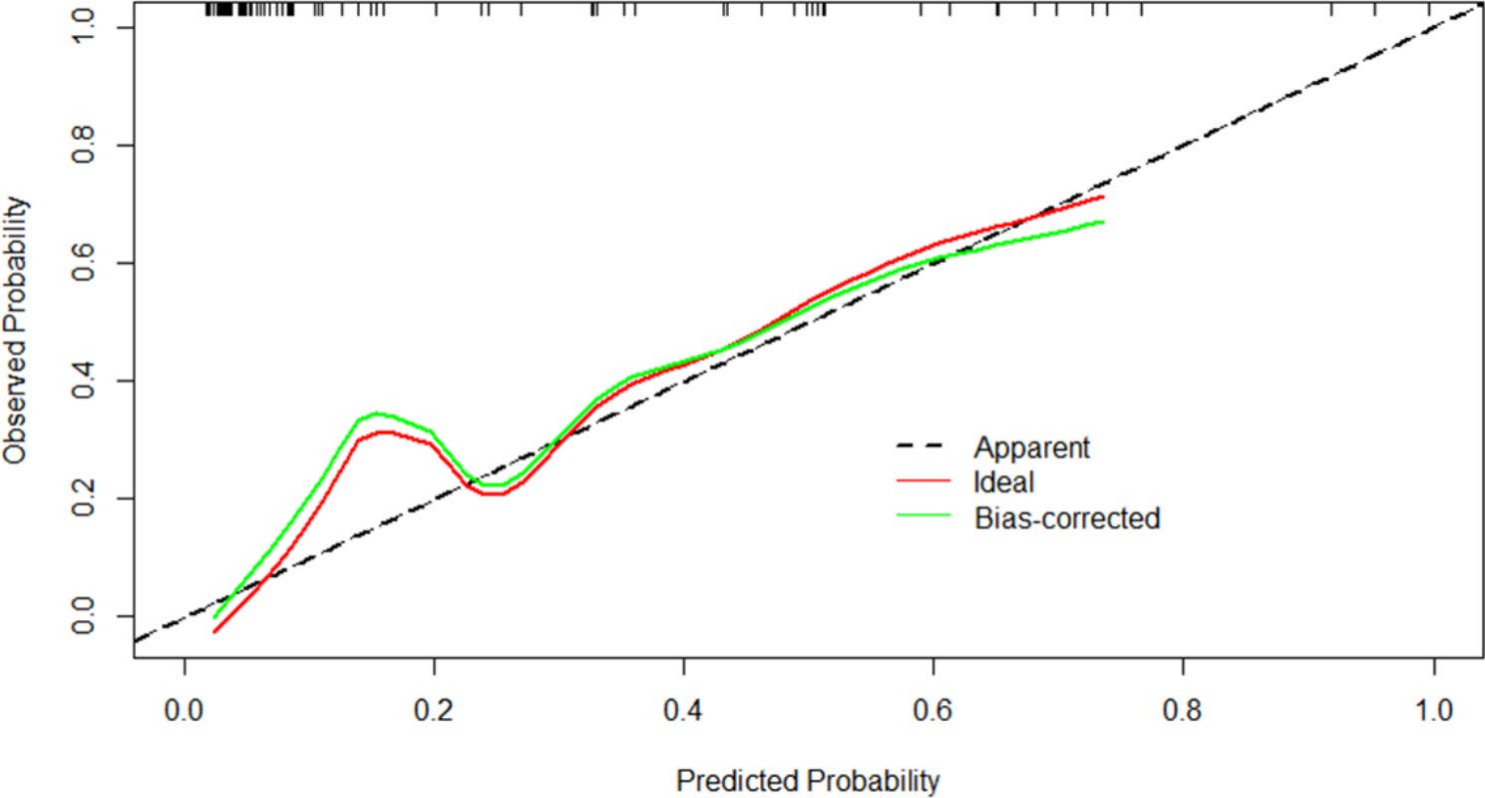
The calibration curve of nomogram in the validation cohort

**Fig. 6:**
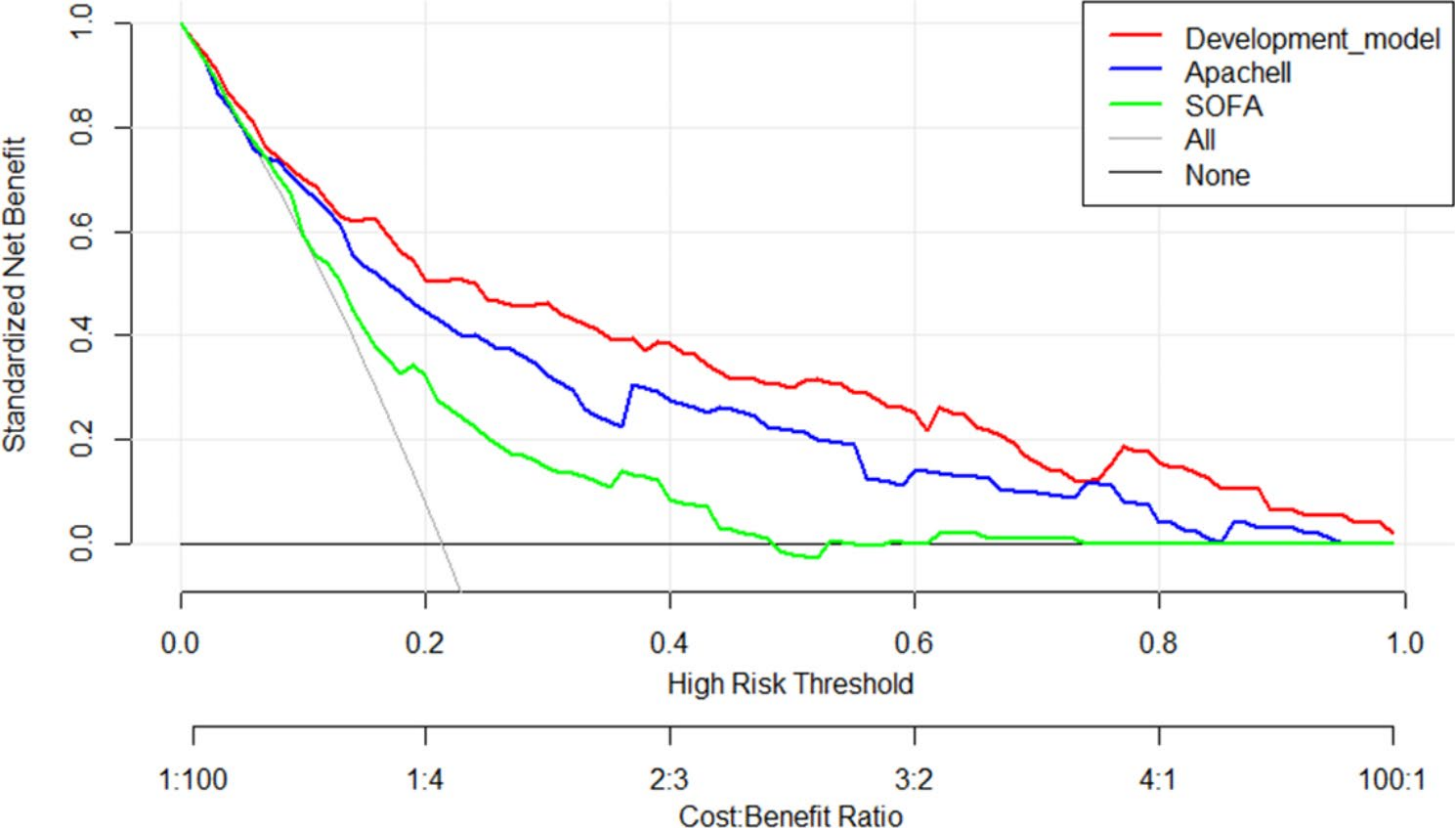
The decision curve analysis of nomogram for the development cohort

**Fig. 7:**
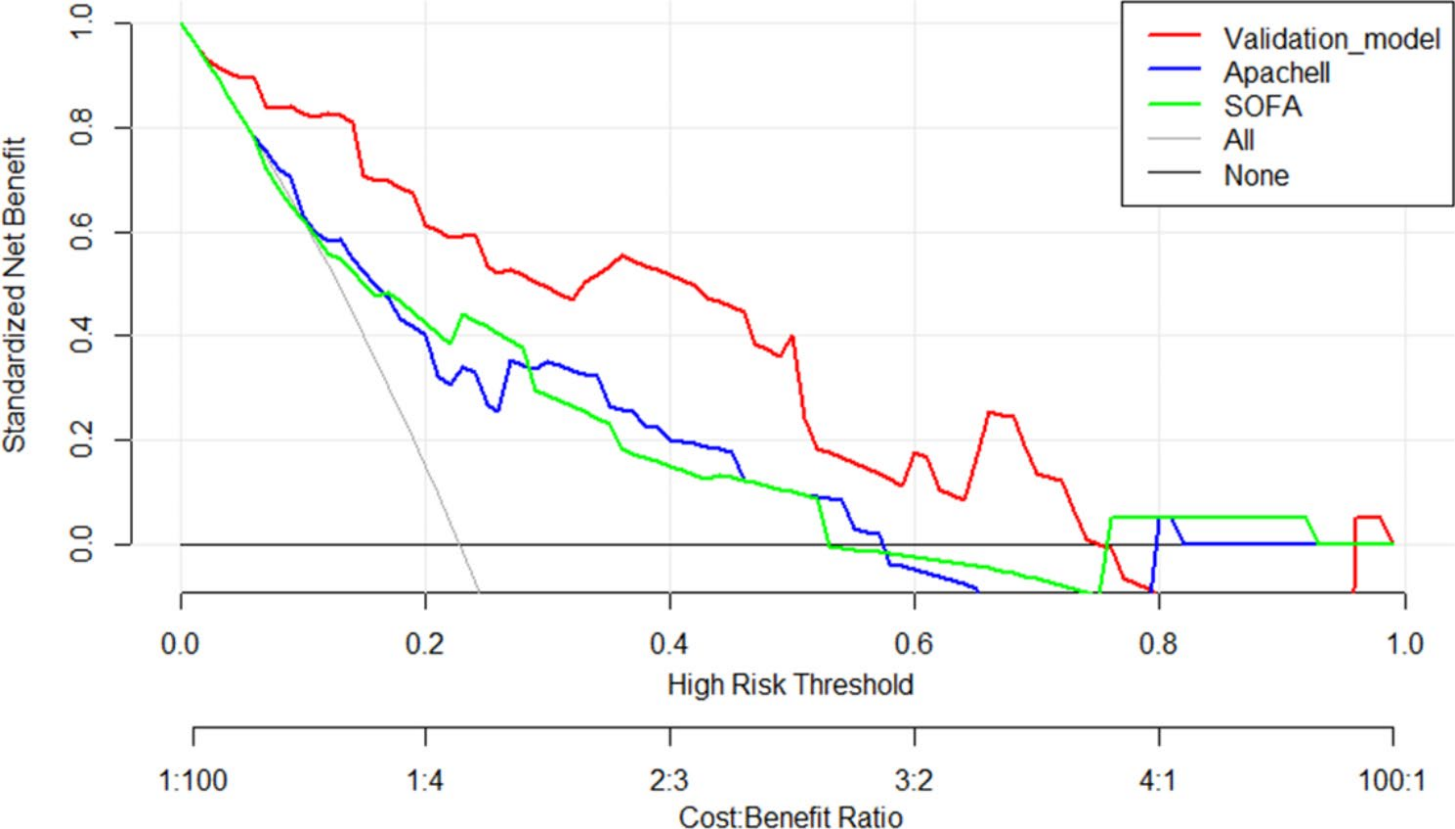
The decision curve analysis of nomogram for the validation cohort

Figures 4 and 5 showed the calibration curves for the development and validation cohorts, respectively, suggesting that the model’s predicted probabilities were close to the observed actual probabilities. The DCA curves for both cohorts were shown in Figs. 6 and 7, with significant net gains for most of the threshold probabilities. Moreover, our prediction model had a higher clinical application compared to APACHE II score and SOFA score.

## Discussion

SCAP is the highest life-threatening type of CAP. The number of patients admitted to the ICU has increased significantly in recent decades, especially among older adults, individuals with comorbidities, and immunocompromised patients, with a high ICU in-hospital mortality rate [15, 16]. Miquel Ferrer et al. reported a 30-day mortality rate of 33% for patients with SCAP on invasive mechanical ventilation in the ICU [17]. The study by Cavallazzi et al. reported an in-hospital mortality rate of 17% in patients with SCAP in the ICU [2]. In our study, the mortality rates of patients with SCAP in the ICU were 21.32% and 22.73% at the two study centers, respectively. This finding is consistent with those of other studies. The high morbidity rate associated with SCAP in patients admitted to the ICU remains a global concern.

Therefore, it was necessary to develop a valuable evaluation system to predict the prognosis in this group. Ideally, such a system would be based on readily available variables in clinical practice. Our study analyzed 455 patients with SCAP admitted to the ICU as the developmental cohort. The final multivariate logistic regression analysis revealed lymphocyte count, PaO2/FiO2, shock, and APACHE II score as independent risk factors, used to establish the mortality prediction model.

As innate immune cells with adaptive immune functions, innate lymphoid cells (ILCs) constitute the body’s intrinsic immune system [18]. ILCs can be classified into ILC1, ILC2, and ILC3 at the molecular level [19]. They are involved in the early inflammatory and immune responses in patients with pulmonary infections, which is manifested as an increase in their numbers [20–23]. In addition, most patients with SCAP admitted to the ICU suffer from respiratory or circulatory failure; therefore, their condition is often complicated by sepsis. It is known that the pathophysiology of sepsis is characterized by an immune cytokine storm in the early stage and immune cell depletion in the late stage [24, 25]. The lymphocytes collected in our study were obtained within 48 h of admission to the ICU, which is just during the cytokine storm phase of sepsis. At this time, the lymphocytes are activated, and later an unfavorable prognosis occurs after a phase of immune cell depletion.

As one of the minor criteria for diagnosing SCAP, PaO2/FiO2 reflects the severity of respiratory failure in SCAP [13]. It has been suggested that the deterioration of PaO2/FiO2 can predict chest X-ray (CXR) abnormalities [26], whereas widespread severe CXR abnormalities are independent factors related to high mortality in patients with CAP [27, 28]. An observational study by Roberto et al. showed that the deterioration of PaO2/FiO2 is an independent predictor of hospital mortality during extracorporeal membrane oxygenation (ECMO) treatment [29]. A cross-sectional study from Italy showed that moderate to severe PaO2/FiO2 impairment was independently associated with a three-fold increased risk of in-hospital mortality in COVID-19 patients [30]. In our study, we collected the lowest level of PaO2/FiO2 within 48 h of admission to the ICU, which suggested that the lower the PaO2/FiO2 ratio, the higher the risk of death.

The APACHE II scoring system has been used extensively for the clinical assessment of critical diseases and as an important guide for the prognosis of ICU admissions. Although APACHE III and APACHE IV scoring systems have been developed and validated for diagnostic accuracy, the classic APACHE II scoring model remains the gold standard for the prognosis of critical care patients in the ICU worldwide [31]. As the APACHE II score does not rely on complex computer algorithms, it is easier to operationalize in clinical practice. Li et al. analyzed clinical data from a cohort of patients with pneumonia in the ICU who participated in a randomized controlled trial that focused on thromboprophylaxis and demonstrated that a higher APACHE II score at admission was one of the factors that influenced survival in these populations [32]. Our study also demonstrated that the APACHE II score is an independent risk factor influencing the prognosis of patients with severe pneumonia in the ICU.

The most common type of shock complicated by SCAP is septic shock, which is one of the main diagnostic criteria for SCAP [13]. Septic shock is associated with high virulence and severe damage to organ function. It is a widely recognized poor prognostic predictor of sepsis [33]. A large multicenter nested cohort study reported an overall mortality rate of 51% in patients with pneumonia-complicated septic shock [34]. Our study also confirmed that patients with SCAP in combination with shock had a worse prognosis.

Nomograms can quantify and visualize every variable’s contribution statistically, eliminating possible confounders among individual variables. In this study, we collected and analyzed the data of patients with SCAP in the ICU to establish a nomogram model. Further, data from another hospital were chosen for external validation. The results show that the model has high predictive effectiveness and provides better discrimination. Currently, there are no uniform assessment criteria to evaluate the risk of in-hospital mortality in SCAP. APACHE II and SOFA scoring systems are used widely in clinical practice. By comparing with their analysis, our prediction model showed higher accuracy and clinical application value. Owing to its high value for clinical application and accessibility of each variable, the model could help ICU physicians better evaluate the prognosis of patients.

The study had several limitations. First, it was a retrospective observational study, and selection bias could not be ruled out entirely. Second, the study only included patients from two hospitals, which may limit its generalizability to other geographical regions and populations. Third, the follow-up period was short and long-term mortality was not observed. In the future, it will be necessary to perform large-sample, multi-center prospective cohort studies to confirm our study results.

## Conclusions

In summary, based on the clinical data of patients with SCAP admitted to the ICU, our study established a visualized nomogram model using lymphocytes, PaO2/FiO2 ratio, APACHE II score, and shock as the central mortality risk factors. The model demonstrated high predictive accuracy, discriminatory power, and clinical usefulness in predicting the risk of in-hospital mortality for patients with SCAP in the ICU. Our study will assist ICU physicians in improving the prognosis of patients with SCAPs.

## Data Availability

The data that support the fndings of this study are available from the corresponding author upon reasonable request.

## Abbreviations

SCAP: Severe community-acquired pneumonia
ICU: Intensive care unit
ROC: Receiver operating characteristic
AUC: Area under the receiver operating characteristic curve
DCA: Decision curve analysis
PaO2: arterial oxygen partial pressure
FiO2: inhaled oxygen concentration
APACHE: Acute Physiology And Chronic Health Evaluation
HAP: Hospital-acquired pneumonia
CRP: C-reactive protein
PCT: procalcitonin
PaCO2: arterial carbon dioxide partial pressure
SOFA: Sequential Organ Failure Assessment;MV, mechanical ventilation.ORs:Odds ratios
CIs: confidence intervals
ILCs: innate lymphoid cells
CXR: Chest X-ray
ECMO: Extracorporeal membrane oxygenation
